# Fructose Malabsorption, Gut Microbiota and Clinical Consequences: A Narrative Review of the Current Evidence

**DOI:** 10.3390/life15111720

**Published:** 2025-11-06

**Authors:** Catarina D. Simões, Ana Sofia Sousa, Sofia Fernandes, Amélia Sarmento

**Affiliations:** 1 FP-I3ID, Faculty of Health Sciences, University Fernando Pessoa, 4200-150 Porto, Portugal; asofiasousa@ufp.edu.pt (A.S.S.); assuncao@ufp.edu.pt (A.S.); 2 RISE-Health, Faculty of Health Sciences, University Fernando Pessoa, 4200-150 Porto, Portugal; 3 Center for Innovative Care and Health Technology (ciTechcare), Politécnico de Leiria, 2414-016 Leiria, Portugal; 4 Instituto de Investigação e Inovação em Saúde, Universidade do Porto, 4200-135 Porto, Portugal

**Keywords:** dietary fructose, fructose malabsorption, gut microbiota, nutrition

## Abstract

Fructose malabsorption is characterized as the incomplete absorption of fructose in the small intestine. Fructose is one of the most common monosaccharides in the human diet. The purpose of this review is to provide an updated overview of insights into the relationship between high-fructose diet, fructose malabsorption, gut microbiota and clinical consequences. Incomplete absorption of fructose causes accumulation in the colon, which leads to fermentation by gut microbiota and abdominal symptoms such as bloating and excessive gas production. Malabsorption may result from exceeding the absorptive capacity of GLUT5 or insufficient upregulation, with incidence increasing with age and higher dietary fructose concentrations. High-fructose diets generally promote an increase in inflammatory bacterial groups such as *Desulfovibrio* and *Deferribacteraceae*, while reducing beneficial Bacteroidetes. These microbial alterations may impair intestinal barrier function, modify short-chain fatty acid profiles, and contribute to systemic inflammation, metabolic disorders, and potentially mental health issues. Animal studies using fructose malabsorption models present inconclusive results regarding the impact of fructose on the composition of gut microbiota. Additional research is essential to fully comprehend the complex relationship between diet, fructose malabsorption and gut microbiota, to develop personalized, effective dietary approaches for managing symptoms of fructose malabsorption.

## 1. Introduction

Fructose is one of the most common monosaccharides that occur in the human diet. Fructose is a ketohexose naturally present in foods in three forms: as free fructose; as a part of the disaccharide sucrose, with glucose in a 1:1 ratio; or as fructans, polymers consisting of fructose units and a terminal D-glucosyl residue [1].

Food high in free fructose includes honey and fruits except avocado, banana, cranberry, lime, lemon, cantaloupe, pineapple, strawberry, mandarin, and orange, which are low fructose alternatives [2]. In addition, fructose may be artificially incorporated into foods and beverages as high-fructose corn syrup (HFCS), a liquid sweetener consisting of a mixture of glucose and fructose. Although the percentage of fructose in a HCFS is not universally defined [3], it often contains 42% or 55% free fructose, with the remainder mainly consisting of free glucose and glucose-oligosaccharides [4]. The typical Western dietary pattern is characterized by an abundance of HFCS, frequently found in processed foods [4].

Sucrose, a disaccharide formed by fructose and glucose units, occurs naturally in sugar cane and sugar beet, honey, berries and fruits. Sucrose is the most prevalent fructose dietary source, which is used in table and processed foods as a sweetener, preservative and to add functional properties [5]. Sucrose digestion is catalyzed by the intestinal membrane α-glucosidase sucrase-isomaltase, into glucose and fructose [6].

Dietary fructans are mostly fructooligosaccharides, present in a variety if cereals, fruits and vegetables [7]. The addition of commercial fructans to processed foods is becoming increasingly common, thanks to their sensory and textural properties, as well as their potential health benefits. Fructans are not digested in the human gastrointestinal tract since it lacks the necessary enzymes to break them down. Therefore, most of them pass through to the colon, where water is drawn into the lumen through osmosis and bacterial fermentation takes place. Fructans are considered prebiotics because they stimulate the proliferation of beneficial intestinal bacteria such as *Bifidobacterium* spp., *Lactobacillus* spp. and *Faecalibacterium prauznitzii* [8,9]. Both fructose and fructans belong to a group of poorly absorbed short-chain carbohydrates and sugar alcohols, the Fermentable Oligosaccharides, Disaccharides, Monosaccharides and Polyols (FODMAPs) [7].

Fructose malabsorption refers to the incomplete absorption of dietary fructose through the mucosa of the small intestine. This leads to fructose accumulating in jejunum and colon [10,11]. Thus, fructose is fermented in the digestive tract, causing abdominal symptoms such as luminal distention, excessive gas production, abdominal discomfort, bloating and motility changes, which affect patients’ quality of life [12]. Hydrogen and/or methane produced by the gut microbiota after consumption of fructose-containing foods or beverages may be detected by breath testing in patients with fructose malabsorption [13,14].

The purpose of this narrative review is to provide updated insights into the relationship between high-fructose diet, fructose malabsorption, gut microbiota and clinical consequences. Nutritional and therapeutic approaches targeting gut microbiota offer significant promise in advancing the personalized management of fructose malabsorption.

## 2. Fructose Absorption

Fructose is passively transported across the small intestinal epithelia through facilitative diffusion. Glucose transporter 5 (GLUT5) has an exclusive affinity for fructose and is primarily responsible for its transport across the enterocyte apical membrane [15,16]. Once in the enterocyte, fructose may be initially metabolized by the fructose-phosphorylating enzyme ketohexokinase (KHK) or transported through the basolateral membrane to the portal vein via GLUT2. At low doses, most of the fructose is metabolized by KHK to fructose-1-phosphate, which is then cleaved to glyceraldehyde and dihydroxyacetone phosphate that enter glycolysis [17,18,19]. Two splicing isoforms of KHK are expressed in the gut: KHK-A, which has a low affinity for fructose and is ubiquitously expressed in most tissues, and KHK-C, which is expressed primarily in the liver, small intestine and kidneys, and has a high fructose affinity. Fructose-fed KHK-A/C knockout mice have impaired fructose metabolism resulting in high serum and urinary fructose levels, which increase further with high fructose intake [17,20]. The conversion of fructose to glucose and other metabolites is reduced by intestinal-specific KHK-C deletion, while fructose transit to the liver and colon is enhanced [21]. Nevertheless, KHK-C knockout mice have a residual level of fructose catabolism in the intestine due to activity of KHK-A, which plays a significant role in fructose metabolism where luminal fructose levels are high enough to activate the enzyme. Dietary fructose up-regulates the expression of both KHK-C and KHK-A [17] and activates the expression of GLUT5 in the proximal regions of the human and rodent small intestine [17,18,19]. GLUT5 may sense luminal levels of fructose, acting as a transceptor [22]. Upon substrate binding, GLUT5 triggers an adaptive response inducing the synthesis of de novo mRNA and enzymes to prevailing nutrient concentrations [15,22]. However, GLUT5 has a low capacity, so excess dietary fructose may overload GLUT5 and prevent complete absorption [23].

Fructose malabsorption occurs when fructose is not effectively absorbed through the small intestinal mucosa, resulting in fructose being delivered to the distal small intestine and colon [11]. While malabsorption may be caused by a reduction in intestinal absorptive surface area, increased intestinal motility or defects in the digestive process [24], it may also be related to limitations in the transport of fructose. Infants and young children with high fructose intake have greater incidence of fructose malabsorption, potentially explained by developmental limitations of GLUT5 expression and regulation [23,25]. The prevalence and severity of fructose malabsorption in humans thus appears to decrease with age [26]. In adults, fructose malabsorption, as evaluated by breath hydrogen, increases in proportion to the dietary fructose concentration [27,28]. As previously mentioned, dietary fructose induces its absorption. However, high fructose intake may overwhelm the carrier’s absorptive capacity, leading to incomplete absorption [23]. Moreover, some adults may be incapable of sufficiently upregulate GLUT5 expression, which may result in elevated levels of dietary fructose surpassing the absorptive capacity of the intestine [23], suggesting that the intestinal capacity to absorb fructose varies between healthy subjects. Consequently, the incidence of gastrointestinal symptoms following fructose ingestion may also vary considerably between individuals [27]. In mice, ingestion of high doses of fructose (≥1 g/kg) exceed the absorption and clearance capacity of the intestine, resulting in fructose reaching both the liver and the colon, where it is utilized by the gut microbiota [29]. Elevated colonic fructose levels in mice are associated with dysbiosis of the gut microbiota and intestinal inflammation [30,31]. *Glut5* knock-out mice demonstrated a diminished capacity for fructose absorption in the jejunum, and lower serum fructose levels [32]. However, fructose transport was not completely abolished, suggesting a potential role for other intestinal fructose transporters.

The occurrence of GLUT2 in the apical membrane of enterocytes has been observed in rodents [16]. GLUT2 is a high-capacity, low-affinity transporter of glucose and galactose that also transports fructose at a ratio of 1:1. It is incapable to transport fructose without glucose. The uptake of fructose in the jejunum of GLUT2-null mice was strongly impaired when they were fed a fructose-rich diet for 5 days. In addition, glucose intake stimulated the recruitment of GLUT2 to the apical membrane in rats [33], supporting the hypothesis that glucose has an enhancing effect on fructose absorption. GLUT5 and GLUT2 could thus play complementary roles in adjusting the absorption capacity of the intestine to occasional or repeated dietary sugar loads. However, it is generally accepted that GLUT2 is primarily found in the basolateral membrane of enterocytes, where it facilitates the transfer of fructose from the cytosol to the portal vein [15]. The significance of GLUT2 and other transporters on the apical membrane remains unclear [32,34,35].

Fructose malabsorption has recently been associated with potential genetic factors. *Glut5* and *Khk* genes are regulated by the carbohydrate response element-binding protein (ChREBP), a transcription factor which controls intestinal fructose metabolism [36]. Thus, lower expression of *Chrebp* may play a role in fructose malabsorption.

The presence of dietary substances that affect fructose absorption may also contribute to malabsorption. Dietary polyphenols, which are bioactive compounds primarily found in fruits and vegetables, interfere with fructose absorption in vitro studies [37,38,39]. The uptake of fructose by the intestinal caco-2 cells was inhibited by several polyphenols, affecting GLUT5 mRNA expression [38]. Quercetin, apigenin and chrysin were the most effective, with uptake being inhibited by 20–25%, possibly acting on both GLUT2 and GLUT5 transporters.

Sensitivity to other FODMAPs may also be experienced by individuals with fructose malabsorption. Dosages of sorbitol and fructose, which are absorbed when consumed separately, may be inadequately absorbed when consumed together and result in gastrointestinal discomfort [40]. Sorbitol is a polyol that occurs naturally in honey, as well as in fruits and vegetables such as apples, pears, apricots, cherries, nectarines, peaches, plums, watermelons, mushrooms and cauliflowers [41]. Thus, a diversity of foods contains both fructose and sorbitol. Moreover, an association between fructose and fructan malabsorption has been recently reported in individuals with irritable bowel syndrome (IBS) [42]. Patients with fructan or fructose malabsorption had a significantly probability of a positive test result for the other carbohydrate [42].

## 3. High-Fructose Diet and Gut Microbiota

The amount of dietary fructose affects how much it is absorbed and the concentration of fructose in the lumen. Consequently, it affects the composition of the gut microbiota. The impact of high-fructose diet on the composition of gut microbiota is summarized in Table 1. In adults, moderate fructose consumption corresponds to ≤50 g/day or ~10% of total energy intake, while high intake corresponds to >50 g fructose/day [43,44,45]. Although the low dose of fructose is undefined, clinical studies have considered small doses of fructose of less than 10 g per meal [45,46].

Numerous studies have observed changes at the phylum level in fructose-fed mice, including a reduction in Bacteroidetes and an increased abundance of Proteobacteria [47,48,49,53]. A 13-week dietary intervention in mice high in glucose or fructose (containing 65% of calories from glucose or fructose and sucrose) induced changes in gut microbiota as compared to mice-fed a normal diet (58% calories in carbohydrates). The abundance of *Muribaculum intestinale* (phylum Bacteroidetes) significantly decreased after both interventions while the proportions of *Desulfovibrio vulgaris* increased (phylum Proteobacteria) [47]. *Muribaculaceae* family members have garnered a lot of interest lately because of their role in preserving host health [54]. These bacteria metabolize both endogenous (mucin glycans) and exogenous (dietary fibre) polysaccharides to produce short-chain fatty acids (SCFA). They can also synthetize vitamins and amino acids, as well as produce rare enzymes characteristic of Bacteroidetes, such as IgA-degrading peptidases and oxalate-degrading enzymes. In addition, *Muribaculaceae* members interact through cross-feeding with beneficial species within the genera *Bifidobacterium* and *Lactobacillus* [54]. On the other hand, *D. vulgaris* has emerged in the last few years as an important pathobiont associated with intestinal disorders such as Inflammatory Bowel Disease, neurodegenerative diseases, and autism [55].

A further intervention, a 12-week fructose-rich diet administered to mice (72% of the energy from fructose), also resulted in increased abundance of the family *Desulfovibrionaceae* (phylum Proteobacteria), in addition to *Lachnospiraceae* (phylum Firmicutes), and *Deferribacteraceae* (phylum Deferribacterota) as compared to other diets cornstarch, branched chain amino acid, soybean oil, and lard that did not contain fructose [48]. While the role of *Lachnospiracea* is still controversial [56], *Deferribacteraceae* may cause inflammatory damage and exacerbate energy metabolism disorders. Fructose-rich diet increased the abundance of the genus *Coprococcus* and *Ruminococcus*, and decreased *Parabacteroides* and *Allobaculum* [48]. Similarly, the numbers of the genera *Coprococcus*, *Roseburia*, *Ruminococcus*, and *Coprobacillus*, in addition to the family *Coriobacteriaceae*, increased in rats fed a fructose-rich diet (0% of energy from fructose) for 8 weeks as compared to controls (0% fructose) [51]. On the contrary, the genera *Bacteroides*, *Lactobacillus*, *Clostridium*, and *rc4-4* decreased. While most species of *Bacteroides*, *Lactobacillus*, *Ruminococcus* and *Clostridium* are commensal bacteria that live in harmony with the intestinal environment [57,58], rc-4-4 is a potential pathogenic genus identified in mice [59].

Another study found that mice fed a high-fructose diet (35% of energy from fructose) for 8 weeks experienced changes in the structure of their gut microbial communities. However, microbial alpha-diversity remained unchanged compared to the baseline levels [49]. The abundance of Bacteroidetes decreased significantly, while the abundance of Proteobacteria increased significantly, in fructose-fed mice. Feeding fructose also led to a significant increase in the abundance of pathogenic bacterial taxa, such as *Deferribacteraceae* (*Mucispirillum*) and *Helicobacteraceae* (*Helicobacter*), in mice. The abundance of *Mucispirillum* is indicative of an early disturbance to the mucus layer of the colonic surface [60]. The study associated a high-fructose diet with neuroinflammatory response in the hippocampus and neuronal loss, as result of gut dysbiosis. Furthermore, although the abundance of bacteria that produce SCFAs, such as *Lachnospiraceae* and *Ruminococcaceae*, increased in fructose-fed mice, fecal concentrations of acetate, propionate, butyrate were significantly lower in the fructose-fed group than in the control [49]. The same study also found that SCFAs can influence gut dysbiosis and reduce the damage caused by fructose to the intestinal epithelial barrier. This suggests that the gut microbiota and their metabolites play crucial role in maintaining the integrity of the intestinal epithelial barrier. This may help to explain the inhibiting impact of SCFAs on hippocampal neuroinflammation and neuronal loss in animals fed a high-fructose diet.

Another study investigated the impact of a sugar- and fat-rich diet in adult mice, with either access to tap water or water containing 30% fructose for 12 weeks and compared to control groups. Although no differences in microbial diversity were identified between the dietary groups, the relative abundance of several bacterial taxa significantly changed [50]. At the baseline, the genus *Bifidobacterium* was equally abundant in each group. However, it increased after the intervention in mice on a Western diet and fructose group, but not in control mice drinking fructose-supplemented water or in Western diet-fed mice without fructose. Bifidobacteria are known to use fructooligosaccharides, sucrose and fructose as a substrate [61], which were incremented in the western-fructose diet. In contrast, the groups supplemented with fructose had lower numbers of Peptostreptococcaceae. Moreover, Western diet-fed mice supplemented with fructose had lower numbers of the genus *Parabacteroides* and *Barnesiella* as compared with the other groups [50]. Although a few studies associate species belonging to *Parabacteroides* with many diseases, *Parabacteroides* spp. may promote host health [62]. Similarly, investigations on the role of genus *Barnesiella* have reported different results. While *Barnesiella* is one of the most abundant genera identified in mouse intestine, its representation in human microbiota is minor. Members of the genus *Barnesiella* restricts colonization of vancomycin-resistant *Enterococcus* in the intestinal tract of mice [63]. In addition, *Barnesiella* may positively affect metabolic disorders through the production of acetate [64]. On the other hand, *Barnesiella* is possibly associated with intestinal inflammation [65,66].

The impact of a fructose-rich diet on the human gut microbiota remains largely unknown. The effects of two sequential short-term diets high in fructose (100 g per day) on the composition of the gut microbiota was studied in female participants. Initially, the subjects consumed a diet abundant in fruit and vegetables characterized by elevated levels of fructose, subsequently followed by a HFCS diet. Both diets were introduced after a low-fructose phase [52]. Changes in the composition of the gut microbiota were observed in different genera. An increase in *Faecalibacterium* and *Anaerostipes* was observed after the fructose-rich diet, whereas a decrease in *Parabacteroides* and *Barnesiella* was observed. In contrast, the HFCS diet led to a reduction in the abundance of the genera *Ruminococcus*, *Faecalibacterium* and *Erysipelatoclostridium*, while *Barnesiella* increased. The amount of fructose in the diet was comparable in both cases, but the fruit and vegetable diet included more fibre than the diet made up of HFCS. Therefore, the observed changes in gut microbiota composition may reflect differences in fibre intake and food matrix rather than to a deviation in the response to elevated fructose ingestion [52]. The genera *Anaerostipes*, *Coprococcus*, *Roseburia*, *Ruminococcus* and *Erysipelatoclostridium* to the phylum Firmicutes are known to produce butyrate. Butyrate is a short-chain fatty acid and the primary energy source for colonocytes [67]. In addition, butyrate has anti-inflammatory properties, modulating both cytokine and regulatory T cells production. It also has a positive effect on intestinal and extra-intestinal homeostasis [67,68]. Therefore, while a diet consisting of fructose-rich fruits stimulates the growth of beneficial butyrate-producing bacteria, a diet consisting of HFCS decreases their abundance, thereby promoting intestinal inflammation and compromising intestinal homeostasis [52]. In another double-blind, crossover design study involving 10 obese subjects, the isocaloric replacement of complex carbohydrates with either 75 g of fructose or 75 g of glucose was investigated over a period of 14 days. No effect on the composition of the fecal microbiota or gut permeability was observed between treatments. *Bifidobacterium* was the only taxon to decrease significantly after treatment in both groups, with no difference between treatments [69]. However, obesity is associated with a dysbiotic gut microbial profile that may explain the lack of impact of the dietary fructose excess in the study participants [70].

The impact of fructose on the dietary modulation of the gut microbiota may be dependent of the concentration of fructose, the duration of the intervention, genetics factors, previous gut microbiota composition profile, and overall dietary pattern. Dietary source of fructose, e.g., fruits and vegetables or sweetened beverages and their components, may affect fructose absorption rate and the modulation of the microbiota composition. The food source and its nutritional and/or chemical composition should be thus considered when addressing high-fructose intake and fructose malabsorption. Future studies should consider host-specific factors, such as genetics and pre-existing dysbiosis, concentration and duration of fructose exposure, other food matrix components, and focus on translating findings to the human gut.

## 4. Fructose Malabsorption and Gut Microbiota

Studies on the impact of fructose malabsorption on gut microbiota composition are scarce and restricted to animal models as summarized in Table 2.

The impact of fructose malabsorption on distal intestinal endocrine function, and the part the cecal microbiota play in mediating the effects of fructose, were investigated using ketohexokinase mutant (KHK^−/−^) mice [66]. KHK^−/−^ mice are a suitable model for moderate fructose malabsorption because they are unable to adapt to elevated levels of fructose in the intestinal tract [17,18]. KHK^−/−^ and wild-type mice were both fed an isocaloric diet containing an additional 20% fructose for 8 weeks. By the end of the intervention, the abundance of the Desulfovibrionaceae family had decreased significantly in the KHK^−/−^ mice, primarily due to a substantial reduction in *Desulfovibrio simplex*. In contrast, families *Coriobacteriaceae* and *Corynebacteriaceae* from the phylum Actinobacteria and *Bacteroidales* from Bacteroidetes increased at the end of the intervention. In addition, *Lactobacillaceae* family, particularly *Lactobacillus johnsonii*, significantly increased in the cecum of the KHK- mice. Since the growth of lactobacilli is favoured in the presence of sucrose and fructose [73], unabsorbed fructose may become a carbon source for the bacteria in the lower intestine. On the other hand, since fructose was not detected in the cecum of the KHK^−/−^ mice and the amount of glucose drastically increased [71], it was possibly converted to glucose in the small intestine [29]. Furthermore, the concentration of propionate in the cecum of fructose-fed KHK^−/−^ mice increased by 50% compared to wild-type mice, possibly due to fructose fermentation via glucose [71]. Propionate is suggested to stimulate the production of cholecystokinin from the enteroendocrine cells, a peptide hormone linked to the gastrointestinal system with several roles such as stimulation of digestion, satiety and gut motility [74].

A different model of fructose malabsorption, *Chrebp*-KO mice, has been recently used to study the impact of a fructose-rich diet on specific GI symptoms, microbiota composition and immune responses [72]. *Chrebp*-KO mice exhibit abnormal fructose-induced glucose transporter expression, making them susceptible to diarrhea and intestinal fructose malabsorption when fed a high-fructose diet [75,76]. Compared to mutant mice fed a control diet, *Chrebp*-KO mice fed a fructose-rich diet (60% fructose) for three days had a decrease in the abundance of the phyla Bacteroidetes and Gamma/Delta Proteobacteria. In contrast, the phylum Actinobacteria, the classes Betaproteobacteria and *Deferribacteres* increased as compared with their wilt-type counterparts who were fed the same diet [72]. Lower taxonomic levels were not analyzed. In addition to changes in the composition of the microbiota, fructose-fed *Chrebp*-KO mice showed decreased IgA synthesis, which is essential for maintaining intestinal homeostasis in the small intestine. There was a reduction in the expression of genes that encode molecules supporting gut barrier integrity.

Fructose malabsorption is associated with low concentration of serum tryptophan, which is implicated in the development of depression [77]. A fructose- and sorbitol-reduced diet improved early signs of depression in fructose malabsorbers and enhanced gastrointestinal symptoms [78]. Bacterial species within the gut microbiota such as *Clostridium* spp., *Bacteroides* spp., *Lactobacillus* spp. and *Escherichia coli* (Gammaproteobacteria) may participate in tryptophan catabolism [79,80]. Luminal fructose may therefore impact the concentration of tryptophan in the colon and the gut microbiota composition. Both phylum Gammaproteobacteria and Bacteroidetes had a significant decrease in *Chrebp*-KO mice fed a diet with 60% fructose. In contrast, KHK^−/−^ mice had an increase in the families *Bacteroidales* and *Lactobacillaceae*; however, their diet only contained 20% of fructose.

Dietary modulation of the gut microbiota in fructose malabsorption may result not only from the concentration of fructose but also the duration of exposure and the presence of other dietary components.

## 5. Clinical Consequences of Gut Microbiota Changes Associated with Fructose Malabsorption

As previously described, a high-fructose diet may change the composition of the gut microbiota, particularly in individuals with fructose malabsorption. If fructose is not properly absorbed in the small intestine, it moves on to the colon, where it is rapidly fermented by the resident gut microbiota. This fermentation process yields substantial quantities of gases, including hydrogen, methane, and carbon dioxide, responsible for the clinical manifestations of bloating, abdominal distension, flatulence, and abdominal pain or cramping. Furthermore, the presence of unabsorbed fructose exerts an osmotic effect, drawing excess water into the colonic lumen, affecting the feces consistency and intestinal motility. Moreover, an excess of fructose in the intestine has been shown to react with incompletely digested proteins, forming advanced glycation end products [76]. These have been associated with metabolic and inflammatory diseases [81,82]. In particular, the intestinal accumulation of advanced glycation end products triggers a local inflammatory response characterized by high levels of pro-inflammatory cytokines and reactive oxygen species which may contribute to the development of inflammatory bowel disease [83].

The profile of SCFA produced by the gut microbiota may also be altered by excessive fructose fermentation. SCFAs are beneficial to gastrointestinal physiology, immune function and host metabolism, as well as the development and homeostasis of the central nervous system. They are key mediators of the microbiota–gut–brain axis and play a vital role in the neurobiological processes underlying depression [84]. Supplementing with SCFAs can improve depressive-like behaviours in animals fed a high-fructose diet [85]. Alterations in SCFA profiles due to fructose-induced dysbiosis could therefore negatively impact mood.

High-fructose intake impairs intestinal barrier function by downregulating the expression of tight junction proteins [86]. The increased permeability allows bacterial components, such as lipopolysaccharide (LPS), also called endotoxin, present in the cell membrane of Gram-negative bacteria, to enter the bloodstream from the intestinal lumen [87]. LPS triggers a systemic inflammatory response that may lead to inflammatory and metabolic disorders [49,88,89]. Non-alcoholic fatty liver disease may be associated with consumption of dietary fructose. Intake of large amounts of fructose caused greater intestinal permeability, bacterial overgrowth and raised LPS levels in the portal blood in mice. This resulted in the release of pro-inflammatory cytokines in liver tissue, activation of Kupffer cells, and increased production of reactive oxygen species by activation of the hepatic Toll-Like Receptor-4, an immune receptor that recognizes chemicals linked to microbial pathogens like LPS [90]. The composition of the gut microbiota is considered an integral component of the gut–liver axis, representing a viable therapeutic target for the management of liver-related metabolic disorders. In humans with obesity given fructose in doses known to exacerbate non-alcoholic fatty liver disease for 14 days, extra fructose did not alter gut microbiota or permeability or result in endotoxemia, in contrast to findings in rodents [69]. However, changes in fecal metabolome were found in this study, suggesting the bacteria might have changed their metabolic activity.

## 6. Personalized Management of Fructose Malabsorption

Dietary approach in fructose malabsorption should consider a personalized intervention, ideally based on the cause of fructose malabsorption and the fructose dietary source. As previously mentioned, the dietary source of fructose, e.g., fructose from fruits and vegetables vs. fructose from sweetened beverages and their components may affect fructose absorption rate and the modulation of the gut microbiota composition. The food source and its nutritional and/or chemical composition should be considered when addressing fructose malabsorption.

Fructose malabsorption occurs frequently in patients with IBS [91]. Indeed, IBS patients have higher prevalence of fructose malabsorption than healthy adults [92,93,94]. Fructose malabsorption symptoms match complaints of IBS patients such as excessive gas production, abdominal distention, pain, bloating, and diarrhea and/or constipation. Improvement of symptoms were reported in individuals with both IBS and fructose malabsorption after following a dietary plan that reduced dietary fructose load and promoted consumption of foods with a balanced glucose to fructose ratio [12]. Furthermore, a fructose-restricted diet implemented in IBS patients for 1 year improved abdominal symptoms, whereas non-adherent individuals to the diet had persistence of symptoms [95]. Although some patients may benefit from restricting their fructose intake, it is unclear what degree of restriction is necessary or how much fructose can be consumed without causing intestinal discomfort [96].

**Figure 1:**
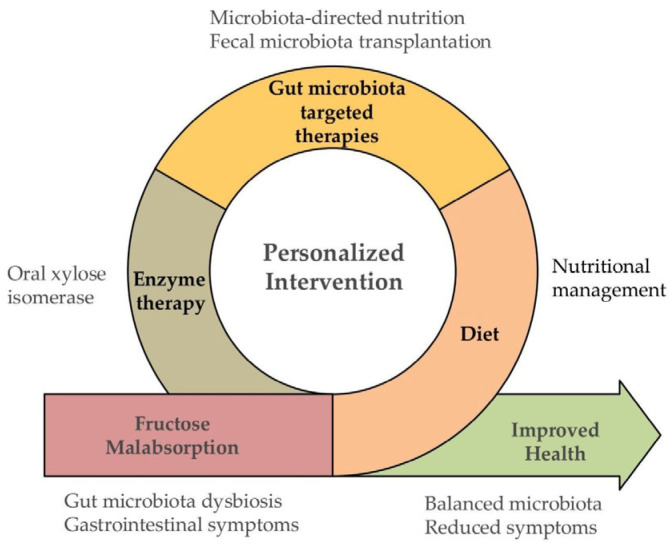
Overview of personalized intervention strategies for fructose malabsorption.

A diet low in FODMAPs has been proposed as a potential therapeutic approach for intestinal conditions such as IBS [7,97,98]. The low-FODMAP diet usually involves severely restricting certain foods for a period of time, after which foods containing individual FODMAPs are gradually reintroduced. This process helps to identify specific sensitivities and enables the customization of a dietary plan in patients with improvement of symptoms after the initial restriction. A recent randomized controlled trial evaluated IBS patients who showed a positive response to the low FODMAP diet. Participants were reintroduced to different groups: 100% fructose, 56% fructose/44% glucose, or 100% glucose, and were given four dose levels (2.5, 5, 10, and 15 g) each day for 3 days. The 15 g dose was well tolerated by most respondents, indicating that patients should be gradually reintroduced to fructose in doses exceeding 15 g to accurately assess their tolerance level [99]. Although a low-FODMAP diet has been reported to relieve abdominal symptoms, possible adverse effects should be considered [100]. Different regions of the gastrointestinal tract may respond differently to individual FODMAPs. Furthermore, the consumption of different types of FODMAPs affects the composition of the gut microbiota in healthy individuals, promoting the growth of beneficial bacteria [9]. For this reason, disease management should be accomplished with the fewest possible dietary limitations to circumvent additional unfavourable repercussions, especially regarding nutritional sufficiency and intestinal microbiota. A personalized approach is essential in the nutritional management of fructose malabsorption symptoms, as schematised in Figure 1. Future microbiota-targeted nutrition approaches could target specific gut microbial strains compromised in fructose malabsorption.

The potential for microbiota-directed therapies, such as fecal microbiota transplantation, to complement nutritional management in fructose malabsorption is a future prospect. Fecal transplantation in rats fed a fructose-rich diet reduced markers of metabolic syndrome, inflammation, and oxidative stress, while restoring the representation of taxonomic units that had significantly increased following the intervention [51]. Furthermore, the first documented case of fecal microbiota transplantation used to treat extensive food intolerances in a pediatric patient with autism spectrum disorder has recently been published [101]. The study reported significant clinical improvements in the management of food intolerances.

Oral administration of xylose isomerase was proposed as a potential therapy for fructose malabsorption due to its ability to convert fructose to glucose. A double-blind, placebo-controlled study demonstrated that oral xylose isomerase significantly lowered breath hydrogen levels and improved symptoms such as nausea and abdominal pain in patients with fructose malabsorption following fructose ingestion [102]. However, further research is required to evaluate the long-term health effects of xylose isomerase and to identify which patients would benefit most from this treatment.

## 7. Conclusions

Dose and concentration of dietary fructose, the type of food or beverage consumed, and the presence of other dietary components affect the absorption rate of fructose and modulate the gut microbiota composition. Although studies addressing dietary fructose intake and fructose malabsorption models present very inconclusive results regarding the impact on the gut microbiota composition, high fructose exposure generally tend to promote the increase in pathogenic and inflammatory bacterial groups. This microbial imbalance may disrupt the integrity of the epithelial barrier, promote intestinal and systemic inflammation, increase the risk of metabolic disease, and possibly influence mental health. Therefore, therapeutic approaches that focus on reducing fructose, restoring the microbiota and supporting the intestinal barrier hold promise for mitigating symptoms and broader health consequences. Microbiota-targeted approaches offer a valuable alternative to simple dietary restrictions by promoting beneficial microbial changes, allowing for more personalized and dynamic management strategies, and improving the quality of life for individuals with fructose malabsorption. More research is essential to elucidate the relationship between diet, fructose malabsorption, and gut microbiota for the development of appropriate and effective dietary and therapeutic interventions.

## Figures and Tables

**Table 1:** Effect of a high-fructose diet on gut microbiota composition.

Study	Intervention	Fructose Content	Gut Microbiota Effects ^1^
Do et al., 2018 [47]	12-week intervention; mice	65% of calories from glucose or fructose and sucrose	↑ *Desulfovibrio vulgaris* (Proteobacteria) ↓ *Muribaculum intestinale* (Bacteroidetes)
Watanabe et al., 2021 [48]	12-week intervention; mice	72% of the energy from fructose	↑ *Desulfovibrionaceae* (Proteobacteria), ↑ *Lachnospiraceae*, *Coprococcus*, *Ruminococcus* (Firmicutes), ↑ *Deferribacteraceae* (Deferribacterota) ↓ *Parabacteroides* (Bacteroidetes)
Li et al., 2019 [49]	8-week intervention; mice	35% of energy from fructose	↑ Proteobacteria ↑ *Deferribacteraceae* (*Mucispirillum*) ↑ *Helicobacteraceae* (*Helicobacter*) ↓ Bacteroidetes
Volynets et al., 2017 [50]	12-weeks; mice; Western-style diet + tap water or fructose supplemented water	Water 30% fructose	↓ *Parabacteroides*, *Barnesiella* (Bacteroidetes)
Di Luccia et al., 2015 [51]	8-week intervention; rats	20% of energy from fructose	↑ *Coprococcus*, *Roseburia*, *Ruminococcus*, *Coprobacillus* (Firmicutes) ↑ *Coriobacteriaceae* (Actinobacteria) ↓ *Bacteroides* (Bacteroidetes) ↓ *Lactobacillus*, *Clostridium*, and *rc4-4* (Firmicutes)
Beisner et al., 2020 [52]	4-week intervention; humans; *n* = 12 Week 1: low-fructose diet Week 2: Fruits and vegetables diet Week 3: low-fructose diet Week 4: High-fructose corn syrup diet	Week 1: 10 g fructose/day Week 2: 100 g fructose/day Week 3: 10 g fructose/day Week 4: 100 g high-fructose syrup diet/day	↑ *Faecalibacterium*, ↑ *Anaerostipes*, ↓ *Barnesiella*, ↓ *Parabacteroides* ↓ *Ruminococcus*, ↓ *Faecalibacterium*, ↓ *Erysipelatoclostridium*, ↑ *Barnesiella*

^1^ Taxonomic level based on each study; ↑ increased or ↓ decreased abundance of the taxonomic unit.

**Table 2:** Effect of fructose-rich diet on the gut microbiota of animal models with fructose malabsorption.

Study	Description	Gut Microbiota Effects ^1^
Zhang et al. [71]	ketohexokinase mutant (KHK)^−/−^ and wild-type mice were fed an isocaloric diet containing an additional 20% fructose for 8 weeks	↓ *Desulfovibrionaceae* ↑ *Coriobacteriaceae*, *Corynebacteriaceae*, *Bacteroidales*, *Lactobacillaceae*
Jang et al. [72]	*Chrebp*-KO mice fed either a control diet or a high-fructose diet with 60% fructose for 3 days	↓ Bacteroidetes, ↓ Gamma/delta Proteobacteria ↑ Actinobacteria, ↑ Betaproteobacteria, ↑ *Deferribacteres*

^1^ Taxonomic level based on each study; ↑ increased or ↓ decreased abundance of the taxonomical unit.

## Data Availability

No new data were created or analyzed in this study. Data sharing is not applicable to this article.

## Abbreviations

The following abbreviations are used in this manuscript:

HFCS	High-fructose corn syrup
GLUT	Glucose transporter
KHK	Ketohexokinase
ChREBP	Carbohydrate response element-binding protein
SCFA	Short-chain fatty acid
FODMAP	Fermentable Oligosaccharides, Disaccharides, Monosaccharides and Polyols
LPS	Lipopolysaccharide
IBS	Irritable Bowel Syndrome
